# Social Assistive Robotics: An Ethical and Political Inquiry Through the Lens of Freedom

**DOI:** 10.1007/s12369-024-01161-x

**Published:** 2024-07-15

**Authors:** Júlia Pareto, Mark Coeckelbergh

**Affiliations:** 1 Florence School of Transnational Governance, European University Institute, Palazzo Buontalenti, Via Camillo Cavour 65, 50129 Florence, Italy; 2 Institut de Robòtica i Informàtica Industrial, CSIC-UPC, Llorens I Artigas 4-6, 08028 Barcelona, Spain; 3 Department of Philosophy, University of Vienna, Universitätsstrasse 7, 1010 Vienna, Austria

**Keywords:** Ethics, Freedom, Healthcare, Human-technology relations, Political philosophy, Social assistive robotics

## Abstract

The development of social assistive robots for supporting healthcare provision faces a lack of an ethical approach that adequately addresses the normatively relevant challenges regarding its deployment. Current ethical reflection is primarily informed by an individual-centered perspective focused on robots’ implications for their end-users and thereby limited to the dyadic human–robot interaction sphere. Considering that this is tightly correlated to the restricted understanding of core ethical concepts upon which reflection stands, this paper delves into the concept of freedom from a philosophical perspective to unfold its full normative breadth for a critical assessment of technological development. By bringing to the fore the political-structural dimension of freedom and, in turn, elaborating the political dimension of technology, the undertaken philosophical approach discloses freedom as a transversal ethical concept for a normative reflection on technology. Thereby, it broadens the scope of ethical attention beyond the sphere of human–robot interaction and turns attention to the so far overlooked structural dimension of human–robot relations. Drawing on conceptions of freedom as non-domination, among others, the paper approaches social assistive robotics and reexamines the terrain of relevant issues for its development. Since freedom is one major issue upon which current concerns revolve, the undertaken analysis substantially enriches the ongoing ethical discussion on social assistive robotics’ implications for human freedom. In this way, this work contributes to going beyond the current individual-centered ethical perspective by laying conceptual grounds for a comprehensive ethical approach to social assistive robotics’ development.

## Introduction

The rising development of social assistive robotics (SAR) [53] as a tool-provider especially for the health care sector poses one of the new calls faced by contemporary ethics. As embodied AI systems that “socially” interact with humans as a means to carry out specific tasks, social assistive robots (SARs) are envisaged in Europe as a resource for professional activities of assistance [13]. That is, as tools for supporting tasks related to aiding people with special needs in different activities of their daily lives, which are held as care delivery either in institutional settings or at homes—e.g., physical and cognitive aid, capacities rehabilitation or maintenance, or even social needs management [7]. Despite the outbreak of ethical attention and discussion on SAR that has come along with this scenario [47], the ethical call remains unanswered. The reason is that the predominant ethical approach to SAR is far from the one required for a comprehensive normative-oriented thinking on technology and its deployment [36].

As disclosed through previous work [34], the ethical perspective from which SAR is primary being approached is an individual-centered one, which focuses on the implications that robots may have for the well-being of humans, specifically for their end-users. This is a narrow perspective, which informs an ethical reflection that is not only mainly engaged with technology implications at a *micro* level of human life, but that is moreover much limited to the sphere of a human–robot interaction (HRI) understood in dyadic terms [48]. Thus, it is the individual life of SARs’ interactants that is at the focus of ethical attention. Given the prevalence of this individual-centered perspective, the dominant ethical approach lacks of a due attention to SAR implications from both the perspective of the care practice in which SARs are (to be) deployed and from the perspective of justice. This is an important shortcoming that renders deficient any ethical approach to technology, and hence to SAR. There are several reasons for this.

First, it implies to overlook the constitutive interrelation between individual well-being, care practices and the sociopolitical activity and structure^1^; in other words, the interconnectedness between the *micro*, *meso* and *macro* levels of human life. By excessively restricting the focus of ethical attention at the individual level—moreover framing it on the dyadic interaction between humans and robots–, the latter is taken as quite disconnected from the kind of care practices and sociopolitical activity that indeed frame the conditions in which individuals’ life takes place, and which thereby have a decisive role on their well-being.

Second—and this is related to the former to a great extent–, it entails to disregard the role of technology in the configuration of that political frame. This is what has already been reported as a neglect of the political dimension of technology [10], that is, of the role that technology plays at the sociopolitical level, not only as an instrument for certain (disputable) ends, but also in the very same shaping of the conditions and structures within which life takes place. Such neglect is typical of an ethics of technology that is either worryingly disengaged from philosophy of technology, or bound to strands of this discipline that may fall short when it comes to attend the political and structural dimension of human-technology relations. In turn, this explains the undue disregard that political philosophy receives within the mainstream normative reflection on technology [43], where conceptual resources of this branch of philosophy are left unused, despite being fundamental for suitably framing the normative questions raised by technoscientific activities [8, 10]. It is crucial, for instance, to ask the questions who decides about SAR and especially for whom they are developed: Who benefits from them and are this necessarily those who are supposed to receive care? Is a democratic approach to SARs possible, given that these technologies are developed by big corporate players? Without such questions, a critical approach is impossible. Furthermore, while approaches such as responsible innovation in social robotics (e.g. [6]) are well-intended with their emphasis on participation and inclusion, they might also shield innovation from a broader political critique that includes a consideration of the (conflicting) interests at stake.

Alongside the individual-centered perspective, there is also a restricted understanding of some of the core ethical and political concepts from which SAR is normatively addressed, such as responsibility or freedom [34], which is mostly taken as a matter of an individual exercise of a human faculty, with disregard for the constitutive relation that such faculty has with the sociopolitical structuring of life. This is a conceptual limitation clearly correlated to the *micro* ethical perspective underlying the current approach to SAR, arguably in a causal sense. Indeed, the narrow way in which such notion is understood impoverishes the scope of issues that are normatively relevant for a SAR development that is truly committed to human freedom, and so to the very same goal of the care practice at which it aims to serve. These missing issues are to be addressed by an ethics of technology that takes into account the political dimension of technology.

In response to these shortcomings of the predominant ethical approach to SAR, this paper unfolds the philosophical notion of freedom and uses it as a conceptual resource from which to ethically (re)examine this specific field of intelligent robotics in a comprehensive way that includes the political dimension.

The paper is structured as follows. The next section delves into the concept of freedom from a philosophical perspective; that is, by considering different accounts that have been developed within this discipline and which point at different dimensions of freedom that are relevant for an ethical reflection about, and an evaluation of SAR technology. Here, special attention is granted to the branch of political philosophy, since it provides key insights in this sense. Drawing upon certain contributions of philosophy of technology, section three tackles the connection between technology and the *macro* level of human life, thereby examining the scope of human-technology relations beyond its “interpersonal”dimension.^2^ This is undertaken as a complementary theoretical groundwork necessary for disclosing freedom as a philosophical notion that leads to a transversal ethical gaze to SAR implications at the three interrelated *micro*, *meso* and *macro* levels of human life. Section four engages in the specific ethical analysis of SAR in the light of the unfolded understanding of freedom. Through it, we map the questions that are normatively relevant for SAR development, and show the limitations of current ethical reflection on this theme. To conclude, section five exposes how the undertaken work is a substantive contribution not only to the ongoing discussion on SAR and freedom, but also to a more comprehensive ethics for SAR, insofar as it redresses the individual-centered perspective towards one addressing SAR development in terms of justice.

## The Political-Philosophical Concept of Freedom

As approached by philosophy, freedom is a concept with a rich and full of nuances scope of meaning, which gathers different constitutive dimensions of this human condition. Thus, turning to philosophy to delve into this idea provides a good ground for any normative reflection seeking to use freedom as analytic lens, as we here intend to.

To unfold the complex and multilayered philosophical idea of freedom, it is important to first recall I. Berlin’s distinction between two different senses of this notion, namely “negative” and “positive freedom” [5].

On the one hand, “negative freedom” refers to the absence of interference, obstacles, or constraints from others regarding one’s own activity. This conception captures an essential dimension of freedom, which has to do with the fact of not being prevented from doing what an agent could do if no one would prevent them to. It is “freedom from” [5], what it is being seized by this notion. Under it, freedom is to be understood as the extent of uncoerced^3^ activity that is available to an agent.

On the other hand, “positive freedom” refers to the presence of control over one’s own decisions and life, that is, to the exercise of self-determination. In this sense, freedom has to do with being the source of the reasons of one’s own actions, in opposition to the fact of being subjected to others’ reasons or to external causes as triggers of one’s doing. A nice formulation of this account is to define freedom as the capacity to choose the reasons for which I choose [40]. Described as “freedom to” [5], positive freedom is thus connected to the philosophical notion of autonomy as the capacity to govern oneself.

Accordingly, taken in its positive sense, freedom is infringed by phenomena like manipulation and paternalism—either be it a classical or a libertarian one [10]—. As an intentional form of immorally influencing the other’s decision-making in a certain direction, manipulation offends against their capacity to act as a (rational) self-directed agent. The same happens with paternalism, for it consists in deciding for another what is in their best interest—and therefrom interfering with their activity against their will (classical paternalism) or attempting to steer their choices towards what is taken to be their best interests (libertarian paternalism), as it is the case of nudging [44]—.

The positive conception resonates well with a conceptualization of freedom from the so-called “capability approach” [32]. The idea of freedom as self-government implies that being free means something more than just not being interfered with one’s own activity. Beyond this, it arguably entails to have certain abilities for self-determination—i.e., for reflecting upon reasons and purposes of one’s own—, and also for effectively taking those as the basis for action.^4^ From a positive account, then, the conditions of possibility of the exercise of autonomy are included under the scope of what being free means. In this sense, as a capacity (to govern oneself), freedom is best conceptualized in Nussbaumian terms of “capability”.^5^ That is, as an *actual* capacity that, as such, is constitutively intertwined with the sociopolitical conditions that make it be a substantive rather than a formal faculty of self-definition—i.e., that make it be possibly exercised or materialized into a corresponding “functioning” [32]—. In light of this, the idea of freedom is sharpened in a fundamental sense, namely in what we could call its political dimension. We will return to this later.

For the moment, it suffices to point out that, under the capability perspective, a new kind of threat to positive freedom can be identified, namely: the phenomenon of “adaptative preferences” [32], which refer to preferences that result from a situation of constraint regarding the opportunity to choose according to one’s one purposes and reasons, and which are therefore preferences built upon an adaptation to such basic limitation.

Besides those featured by the negative (non-interference) and positive (self-mastery) conceptions, there is another key dimension of freedom, which is the one highlighted by the contemporary republican conception of “freedom as non-domination” [37]. Freedom here refers to the absence of domination by others, where domination is understood as power, meaning the actual capacity of agents to arbitrarily interfere in certain choices that another is in a position to do. That is, the capacity of agents to intentionally obstruct the other’s choice situation (interference) at their discretion, disregarding the interests and opinions of those affected (arbitrary). In this republican sense, then, freedom has to do with not being subjected to a *potential* arbitrary interference.

“Freedom as non-domination” thereby differs from the negative conception of freedom in that it is not (primarily) the absence of a de facto interference, what determines the extent of one’s freedom, but rather the enjoyment of a certain (relational) status in which one is not exposed to such coercive phenomenon. Indeed, domination can take place without interference, as it happens when an agent is permanently exposed to arbitrary interference due to its status within a certain social structure. To clarify this, it is useful to recall the relationship slave-master that Pettit takes as an exemplary case of domination [37]: whether the master does actually coerce the slave or not, the latter is unfree because of being exposed to the possibility of an arbitrary interference on the part of the master. In turn, interference can occur without domination, as it is the case when the coercive act does not respond to the exercise of a structural power asymmetry.

The republican approach to freedom as a matter primarily of no subjection rather than of no determination—in other words, its focus on power (a *potentia*) instead of on interference (a fact)—leads to a refined understanding of freedom as a relational condition. Otherwise said, as a capacity that is defined by the network of intersubjective relations in which the agent is placed [31], as stressed by philosophy feminist critiques of the traditional atomistic conception of autonomy, which have led to reconceptualize it as "relational autonomy" [29]. In turn, this points at freedom as inherently linked to the sociopolitical structures under which such (power) relations are framed—i.e., to what we could refer to as a structural dimension of freedom—.

In that respect, a deeper reading of freedom as absence of domination is yet possible if understanding domination not only in the liberal republicanism sense but also in a Žižek/Marxian one, closer to the concept of “objective violence” [55]. That is, in terms of “structural domination”, and not only of “intersubjective domination”,^6^ as we shall name them.

Under the liberal republican view, domination is understood as possibly exercised only by agents (either personal or collective) to other agents, but not by systems or networks [37]. It is in this sense that the liberal republican account of freedom as non-domination rests upon an idea of intersubjective domination. This rules out sociopolitical dispositions such as the institutional, economic and symbolic/ideological order as agents of domination. Žižek’s conception of objective violence, though, helps remedy this, since it discloses another form of domination which is not attributable to particular individuals but rather precisely to that structural order. Unlike that kind of violence perpetrated by particular identifiable individuals (subjective violence), objective violence is impersonal, in that it is not traceable to specific subjects and intentions, but it comes from the same symbolic and structural order on which (intersubjective) power relations are grounded. This definition, insofar as it introduces the notion of an impersonal power of structures, contributes to disclose domination as taking place in a structural and not only an intersubjective form. As we shall see in Sect. 4, such conceptualization of domination introduces important nuances for thinking about the political relevance of technology, as it places the socio-symbolic order (in its linguistic, ideological and structural form) within the scope of consideration of technological development.

The so far examined philosophical conceptions of freedom disclose a key feature of this human capacity, which is its political-structural dimension. From a philosophical perspective, far from being a matter of individual capacity or exercise, freedom is constitutively linked to the sociopolitical framework within which it is (to be) exercised. Meaning, there is an intrinsic relation between freedom and the sociopolitical activity and structure in which individuals’ life takes place. Non-interference, self-determination (or autonomy) and non-domination—both in intersubjective (republican) and structural terms—: all these senses of freedom—which we take as constitutive of a comprehensive understanding of this idea—point at this relation.

Whereas this has become evident from the non-interference and non-domination perspective of freedom, a brief remark may be useful to clarify the pivotal role of political activity in its depth regarding positive freedom. As contended, freedom requires certain conditions that make it possible for an agent to reflect upon their own reasons and to lead their actions as a result of this exercise—thereby enabling to pursue autonomy as effective freedom—. This appeals to political activity in that—borrowing Nussbaum’s terminology—, these conditions have to do with fostering “combined capabilities”, which are an agent’s set of real opportunities to choose and act—and so the set of feasible “functionings”^7^ that they can achieve in different spheres of life—[32]. Since this set results from a combination of both internal abilities^8^ (which are developed in interaction with the environment) and the political, social and economic framework in which the functioning associated with those can be actually exercised, freedom is clearly linked to politics. Regarding those (combined) capabilities, it is worth reminding that Nussbaum defines a list of ten central ones that must be protected up to a certain threshold level for a decent human life. Whereas discussing Nussbaum’s commitment to specific capabilities and undertaking an exhaustive analysis of SAR from her capability approach would be beyond the scope of this paper, three of these central capabilities are indeed key with a view to a SAR development committed to human freedom, namely: “practical reason”, “affiliation” and “control over one’s own environment”.

The political-structural dimension of freedom disclosed by the philosophical approach is of paramount importance for a normative reflection on freedom and its associated challenges or threats. Given that freedom has a dependence on the world and basic sociopolitical structures in which it is exercised, reflecting upon freedom demands, in turn, to critically reflect upon those former. This expands the scope of reflection on freedom beyond the *micro* or individual level of human life, and places the *meso* and especially *macro* spheres of human activity under analysis. Thinking about freedom issues necessarily entails to reflect upon the capabilities, power relations and social structures that condition and frame the exercise of this human capacity. Therefore, concerns on freedom must involve attention to justice, not only in distributive but also in structural terms.

## Yes, but… What Does Technology Have to Do With It? On Human-Technology Relations

Now, when it comes to reflecting upon technology from ethics, a conceptual issue arises which is key to determining the normative relevance of that political-structural dimension of freedom regarding technological development: What does technology have to do with such dimension of freedom? In which sense is there a connection between technology and the sociopolitical frame within which freedom is exercised? Which role does technology play regarding the sociopolitical structuring of human life that decisively conditions freedom’s development and exercise? In brief, the issue here revolves around what can be conceptualized as the political dimension of technology.

The reason why clarifying this issue becomes essential is a matter of argumentative consistency. That freedom is constitutively interrelated with the sociopolitical framework within which human life takes place—as highlighted by a philosophical approach—does not necessarily lead to turn attention to such framework as part of an ethical approach to technology. Unless, technology does have a role in its configuration, as it has indeed been disclosed by certain strands of philosophy of technology.^9^

How is, though, that idea of a political dimension of technology exactly to be understood? In what sense can technology be said to take an active part in the sociopolitical arrangement of human life?

A first attempt to provide an answer would be to recall the instrumental character of technology. In its constitutive dimension of being a means for certain ends, technology may serve as an instrument for specific political purposes, either be it or not deliberately conceived for those in the first place, and, more interestingly, either be these purposes realized through the immediate use of artifacts or through their very same design features. To illustrate this latter way in which technologies are (instrumentally) political, it suffices to think about the well-known case of R. Moses’ bridges over the parkways on Long Island. The intentionally-devised architectural characteristics of the bridges prevented low-income classes and racial minorities to reach Jones Beach [54], thereby imposing a certain social order characterized by inequality in terms of substantive opportunities. The instrumentality of technology, then, for it places artifacts as possible means of settling a specific order of social relationships within human collectives [54], provides a ground to talk about a political dimension of technology.

For the instrumentality of technology to be a proper ground to attribute a political dimension to technology, though, that instrumental character is not to be naïvely understood as morally neutral. Against the modern conception [16], technology is not, in itself, a value-free means for reaching certain ends. This is so for at least two reasons.

On the one hand, because as the product of a specific sociohistorical context, technology always entails a particular cosmovision [22]. Technologies arise from a specific way of understanding and relating to the world and to others—in virtue of which these are precisely conceived as tools—. In this sense, insofar as it responds to a particular commitment to reality on the basis of anthropological, epistemic and political assumptions, technology is, from its very same conception, axiologically charged. Contemporary critical theory of technology has made a point of this by stressing the social character of technology, that is, the interconnection between social meanings (and relations) and functional rationality [17].

On the other hand, because technology is not only the product but also the source of a particular way of thinking about and being in the world, as stressed already from the classical phenomenological analysis of technology. Technology frames the way in which the world appears to us and thereby also conditions how we understand and relate to it [21]. As it were, technologies open up the world in certain (rather than other) forms. Postphenomenology has refined this insight by pointing at the mediating role of technologies in human-world relationships [41], both in hermeneutic and existential terms [23, 49]. Technological artifacts actively shape how reality appears to and is interpreted by us, as well as how we act and organize our lives. On the grounds of these two main dimensions of mediation—mediation of perception or experience and mediation of action or praxis—, technologies can be contended to shape moral decisions and, thus, to mediate morality too [51]. Insofar as they provide access to reality in certain ways rather than others, and invite or inhibit certain actions rather than others [51], then, technologies are far from neutral instruments.

This phenomenon of “technological mediation” is relevant for an accurate account of the political dimension of technology. Although this—with the exception of some more recent attempts [52]—has been left quite unexplored by the very same postphenomenology [15] [25] [50] [9], understanding technologies as mediators of human-world relations opens up a further sense in which technology has a constitutive role at the sociopolitical level of human life, beyond that one attributable to its instrumental dimension. Ultimately, this has to do with a double level of technological mediation. Technologies’ role in framing humans’ hermeneutical and existential stance with and within the world applies both for the *micro* and *macro* level of human life. As pointed at by latest postphenomenological studies, technologies do not only influence the user’s perception and experience, and thereby their decisions and actions, but they also have a constitutive role in cultural and moral frameworks of interpretation, and in social practices [26] [52]. It is in this sense that, from a postphenomenological perspective, human-technology relations are contended to have a political dimension [52], which goes beyond the more strictly instrumental dimension of such relations—that is, beyond the politics that these relations may entail in virtue of the instrumental use of artifacts towards certain ends—. Despite a more specific analysis on the praxis side of technological mediation at the *macro* level would still be in order to thoroughly delve into this, the connection between technological mediation and the politics of technology seems undeniable. By shaping human experiences, margins of action and situations of choice, specific social and thus power relations are also framed and organized through technologies. Consider for example birth control pills, which, by disconnecting sex from reproduction, changed women’ and also homosexuals' relational standing within society. But also SARs are likely to change power relations, as we will show below. This connects to critical theory’s understanding of technologies as frameworks for ways of life, rather than as mere tools [16].

Notice that technological mediation also allows to make better sense of the phenomenon of the non-intentional political implications of technology, so well highlighted by Winner through different case examples [54]. That is, the fact that technology has a configurating role in the social order beyond that which may be intentionally sought—i.e., beyond the one that it has in virtue of being purposedly used as an instrument for arranging power and authority.

Drawing upon contemporary philosophical insights, then, it is not only as (non-neutral) instruments that may serve specific political ends and distribution of power, but also in virtue of their mediating character, that technologies do have a political dimension.

Such account of the politics of technology refines the understanding of human-technology relations in a way that is key for a normative reflection and technological development, for it discloses what we will call the structural dimension of such human-technology relations. Beyond an “interpersonal” level of human-technology relations, in which humans relate with (mediating) technologies in terms of use or of interaction with artifacts, the relations between humans and technologies are also of a structural kind. That is, technologies do enter in relation with humans—thereby having implications for human life—not only in virtue of human direct involvement (usage or interaction) with them, but also more indirectly in virtue of their active coshaping of frameworks for life. Thus, humans relate with technologies not merely as users or interactants with those, but as subjects within technologically coshaped social structures. In next section, we will show the extent to which this dimension calls for a reconsideration of the scope of current ethical discussion on the specific technoscientific field of SAR.

## (Re)Examining SAR in the Light of Freedom

In the light of the philosophical idea of freedom, which accounts for the connection between this capacity and the sociopolitical framework of human life, and taking into account the configurating role that technology plays at that *macro* level (i.e., the political dimension of technology), let us now turn specific attention to social assistive robotics (SAR) to (re)examine the normatively relevant issues that arise regarding its development. As advanced, in Europe such development envisages technologically contributing to healthcare provision, both the one delivered in institutional (daycare centers, long-term care homes or hospitals) and domestic settings. The rationale of such technological policy is sustained by the need of responding to the “nursing crisis” of public healthcare systems in the face of a growing elderly population, and is notably in line with by a dehospitalisation healthcare logics that converges with a European commitment to an active ageing and independent living [30]. Recent illustrative instances of SARs are the socially interactive AI robotic system that helps dementia patients in cognitive training exercises [2,3]; and the social assistive robot Misty II that helps dependent elderly that live alone with their needs in domestic daily life [1].

A previous contextualization is in order. As identified through a critical literature review [34], in the landscape of scholar ethical reflection on SAR, freedom—referred to either (and sometimes indistinctly) as human “freedom” or “autonomy”—is one of the main issues upon which concerns do revolve. From a hermeneutic-teleological perspective on the practice which SARs aim to serve—imperative for an ethical reflection on technology [35]—, this is a reasonable focus of reflection. Indeed, as part of the practice of care, assistance is ultimately linked to the provision of certain conditions for a meaningful and thus capable life. However, the mainstream understanding of freedom in current ethical discussion proves to be a quite narrow one, in that it overlooks some significant dimension of this human capacity. Especially, the political-structural dimension stressed by philosophical conceptualizations, as a revision of current freedom-related concerns shows. So, whereas approaching SAR challenges from the perspective of freedom is not a novel move—hence that we speak of a *re*examination—, doing it from the philosophical notion of freedom is indeed, since it maps a new terrain of freedom-related issues that so far remain out of the scope of ethical consideration.

So far, the problems that SAR raises for human freedom are mainly thought in individual key. They mostly concern the hampering of individuals' exercise of this agency-related faculty, specifically that of robots' end-users. Indeed, were we to summarize the different ways in which SAR is considered to challenge freedom in current literature [34], we could talk about two main kinds of endangerments to freedom associated with this branch of robotics. On the one hand, the interference with users’ action and decision, basically in virtue of robots’ technological autonomy—through which, even if it is in the name of the user’s well-being and health, these AI systems may restrict humans’ courses of action (classical paternalism case)—. On the other hand, the exploitation or fostering of human autonomy’s vulnerabilities, in terms of both functional independence and chiefly Kantian self-determination (i.e., “Can I do ‘x’ or not by my own?” and “For which reason do I choose to do ‘x’ or not?”, respectively). Whereas challenges to independence relate to the risks of an inappropriate assistance leading to users' loss of capacities alongside a dependency on technology (e.g., the user was previously able to eat alone, but stopping exercising the functioning has weakened their capacity to do so), challenges to self-determination mostly relate to the risks of socially interacting with the robot, such as exposure to manipulation, emotional attachment and improper decision-making delegation to the artifact. Only on few occasions is SAR endangerment to self-determination argued in terms of the violation of users' capacity to live according to their own reasons that robots' implementation entails, inasmuch as it is grounded on interests alien to end-users’ ones.

Such landscape of concerns thus reveals that problems for freedom are primarily and almost exclusively being set as problems for the freedom of individuals with whom robots interact, insofar as they are mostly taken as a matter of direct use or interaction with robots, i.e., as problems that arise within the context of (dyadic) human–robot interaction (HRI).

From a philosophical viewpoint, this is representative of a predominant micro perspective of freedom as an individual exercise of a human faculty, disconnected from the kind of practices, power relations and sociopolitical structures in which the latter is exercised—i.e., the *meso* and *macro* levels of human life—. Indeed, drawing upon the multilayered philosophical idea of freedom, current concerns appear to be (more or less exhaustively) informed by the two dimensions of freedom related to the negative and positive conceptions, namely: non-interference and self-government or autonomy. This explains that freedom issues relate to the fact that robots may coerce the courses of action of their users as well as infringe upon their ability to act as rational self-directed agents. What remains out of the scope of attention, though, is the political-structural dimension of freedom, so well stressed by a capability-approach version of positive freedom and by contemporary conceptualizations of freedom as non-domination, which account for the relationality of autonomy. And, with it, the political dimension of technology, which evinces the flaw of an exclusive focus on the “interpersonal” level of human–robot relations.

In the light of these other defining senses of freedom so far overlooked in ethical discussion, more issues appear as essentially related to SAR implications for human freedom. Let us now examine this, beginning with freedom as non-domination.

First, from the (liberal) republican understanding of freedom as a relational condition of no subjection to potential arbitrary interference, SAR implications for human freedom concern the kind of intersubjective (power) relations that are fostered by such technological development—on the grounds of which individuals’ autonomy is actually delimited as a capability^10^.

Of course, this implies the relations between those particular agents involved in the care practice in which robots are implemented. Given the asymmetry of power that care relationships entail, this is a key topic regarding the development of technologies for assistive contexts. SARs’ introduction may reframe power between care-receivers and (formal or informal) caregivers in a way that raises the exposure to arbitrary interference—realizable either by robots or humans in charge—within care relations. For instance,^11^ the data gathering that SARs may undertake in their daily domestic assistance could expose elder end-users to paternalistic interferences by their caregivers (doctors or relatives) to a greater extent than before. Sadowski et al. [42] talk of ‘Big Mother’ as a system that, under the guise of care, manages, monitors, and marketizes domestic spaces and practices, and in this way controls people by structuring their behaviour and limiting their choices. Furthermore, SARs implementation may place new agents of power in former relational networks of care, which may bring with new modes of subjection to interests alien to the care practice defining ones. For instance, inasmuch as private technology companies become part of such networks, the power between the public healthcare sector and private companies may be rearranged in terms of the defining of healthcare models.

Thinking in these non-domination terms also applies to relations with and between “nonnurturant” [14] care agents, i.e., workers that carry on the nonrelational tasks of care (otherwise called “dirty work”), such as cleaning, food preparation or service. SARs’ introduction may sharpen existing instances of domination in labour relations around that part of care (e.g. rising “nonnurturant” workforce’s vulnerability to exploitation). At the same time, it may bring with new professional categories of “nonnurturant” care (e.g., related to the development, functioning and maintenance of the robotic AI systems) that may also reshape power in such terms, even at a global level. For example, relations of exploitation between tech companies and workforces of data labeling for the training of AI systems [11] may also arise from SAR development.

Notice that an approach to SAR in the light of the (liberal) republican account of non-domination thus points at a reflection in structural key. That is, it introduces the sociopolitical structure of intersubjective relations that robotics help configure within the scope of ethical consideration, thereby taking seriously the social dimensions of personal autonomy [28]. Thinking about relations implies attending them also in broader terms of structural networks: Which particular social order is maintained or created through SAR development and implementation?

Second, from a Žižek/Marxian understanding of freedom as non-domination, SAR implications for human freedom concern the socio-symbolical and structural order that SAR helps constitute—which ultimately grounds the relational standing of subjects—. In the light of structural domination, at least two main areas of consideration arise regarding SAR development: an ideological and a more infrastructural-related one, that is, having to do with the mechanisms of human functioning.

On the one hand, comprehending that the socio-symbolical order functions as an (impersonal) form of power—in that it molds situated subjectivities—demands to reflect upon the kind of anthropological and sociopolitical assumptions and narratives that SAR entails and fosters. Which conceptions of the relational selves does robotics promote or reinforce through, for instance, the design and the roles assigned to robots? SARs’ appearance-related traits such as gender, colour or the AI-systems’ voice [46], altogether with the role that these artifacts endorse, may strengthen social stereotypes, bias and inequality on morally irrelevant grounds such as gender or race. Which comprehensions of the meaningful life, vulnerability, autonomy and other care-related ideas are set through SAR development, thereby constituting the socio-symbolical background on which individuals stand in relation to themselves and others? For instance, the kind of assistance that SAR is conceived for, insofar as it is exclusively aimed at replacing individuals’ (disfunctioning) abilities, may reinforce capacitism by sustaining the medical-rehabilitation model of disability to the detriment of the social one [4].

On the other hand, in the light of structural non-domination, freedom not only relates to the socio-symbolical order, but it also has to do with what we will here refer to as the infrastructure of human functioning. In this sense, domination has to do with the framing of human functioning into certain restrictive patterns. On one side, this framing may well come as a self-imposed regulation of one’s own doing triggered by the kind of “panopticism” [18] that SARs entail. Picking up on the issue of SARs’ potential data gathering in domestic environments, for instance, not only end-users but also the people entering their private sphere (e.g., relatives or other visitors) may modify their behavior, conversation or interaction in the face of the surveillance under which SARs’ use may put them. On the other side, though, structural domination in this sense is a more even complex issue that directly links to the phenomenon of technological mediation or, otherwise raised, to the fact that technology constitutes frameworks for certain ways of life. In light of this, SAR implications for human freedom have to do with how this technology delimitates the margins of conversion of capacities to functionings. Which kind of structural order of functioning does this technology imply, and does it restrict human capabilities? Does SAR structurally frame capacities to do and be in a way that limits the range of available options rather than expands them? An enlightening question to land this matter: Are there alternative options to turn our capacities into functionings outside the conversion order that SAR technology provides? SARs’ implementation in elder-care facilities to assist them in daily tasks such as dressing or eating could for instance configure an infrastructure of functioning that restricts the elderly’s alternative ways of operating towards these ends.^12^

In turn, this connects to the third sense of freedom left insufficiently addressed within the mainstream ethical discussion, despite the notable work existing on technology from a capability approach already [24] [33]: positive freedom or autonomy understood in capability terms, that is, as an actual faculty to conduct one’s life according to one’s reasons and purposes. Under it, SAR implications for human freedom concern the set of opportunities for being and doing that robotics helps configure, not only in terms of individual capacities but also more structurally in terms of “functionings environments” [45]. Let us unpack this.

In the light of such account of freedom, the question is the extent to which SAR does stimulate, preserve or amplify human possibilities to be and do, and for whom. Within assistive contexts, this first relates to Kantian autonomy, thus concerning the capacity to choose the reasons for which one chooses [40], in this case that of end-users. In this sense, a basic instance of freedom’s infringement is SARs’ use on the grounds of an “adaptative preference” [32], as it would be the case had an elderly person decided to introduce this assistive AI technology at home not because this adjusts to their own reasons and purposes, but because of being this a preference resulting from a lack of alternative options at disposal (“Better this rather than nothing” would be then the underlying reasoning of such act of consent).

A brief remark is in order here: against what seems to be the mainstream tendency in current ethical reflection, understanding care properly as a relational practice—as for instance proposed in feminist approaches to technoscience [12]—could imply that not only end-users and healthcare professionals but also patients’ relatives should be considered when reflecting upon SAR development in the light of positive freedom.

At the same time, committing to freedom as an (effective) capacity to self-determination demands SARs' development and implementation to serve certain Nussbaumian central human capabilities. Namely, the architectural capabilities of “practical reason” and “affiliation”, as well as of “control over one’s environment”, insofar as they are essential to conduct one’s life according to one’s own reasons and purposes. In turn, this leads us to a crucial related issue: to distinguish well between capabilities and functionings^13^ regarding the goal of assistance provision—and, thereby, what SARs should ultimately aim at supporting—. Indeed, with a view to freedom, there is a decisive distinction between enabling capabilities (and thus the opportunity to exercise them or put them to function) and imposing functionings without fostering capabilities. For example, imagine that SARs were introduced in an eldercare facility to assist physically disabled residents to get dressed, but these end-users had no choice to decide why or why not (so when and in what circumstances) to use the robot. Imagine that they were instead by default daily forced to rely on the robotic-assistance for the sake of the timetable dynamics that is proper of the formally administered round of life of the so-called “total institutions” [19]. In this case, it would be the end-users' functionings but not their capabilities, that SAR would foster. This would infringe upon freedom as (actual) autonomy, for the robots’ use would neither respond to the user’s capacity to reason on the reasons for action (practical reason), neither promote the opportunities to exercise it.

Sticking to distinction between capabilities and functionings, the questions on SAR development arising from the capability perspective are not only to be filtered in individual, but also in structural terms. The focus is here on the kind of “functionings environments” that SAR shapes, in the sense of the kind of infrastructural dispositions for converting individuals’ capacities into functionings that are (co)created by SAR. Does SAR expand rather than restrict the ways of realizing diverse capacities, and thus the range of potential functionings? Notice that this renders the capability-issues intertwined with the second kind of structural domination-related issues stressed above, by linking the former to the conversion order or infrastructure of human functioning that technology brings with it. Yet, it also points to a further and broader issue, in that it calls to reflect even broader upon the material socio-technical configuration that SAR provides, and about whether it is responsive to “social vulnerability” [27]. For instance, SAR focus on promoting personal autonomy by means of assisting in disabilities could overlook the link of human autonomy to social vulnerability, and thus the role that assistive technologies could undertake regarding the provision of material facilitating environments for an egalitarian conversion of diverse capacities into functionings (Table 1).

**Table 1:** Overview of concepts and freedom-related issues for SAR development

Philosophical accounts of freedom	Types of normatively relevant issues for SAR development	Examples of potential violations in SAR use and development
Negative freedom	Non-interference with human courses of action	The robot coerces users’ actions and decisions on paternalistic grounds
Positive freedom	(Human) Self-determination (as Kantian autonomy)	The robot influences users’ decision-making in a specific direction
Capability approach	Effective capacity to self-determination (e.g. conditions of possibility for its exercise)	SARs’ use responds to an adaptative preference
Non-domination	Intersubjective power relations that SAR helps configure	SARs’ use expands care receivers’ exposure to arbitrary interference by caregivers
	Socio-symbolical order that SAR entails and fosters	Grounding SAR development on a medical-rehabilitation model of disability overlooks the social dimension of vulnerability
	Structural order of human functioning that SAR helps configure	SARs’ implementation in elder-care facilities narrows the range of ways of converting capacities to functionings

In sum, in the light of a philosophical understanding of freedom, SAR challenges for freedom concern not only the individual exercise of such an agency-related capacity, but also the basic sociopolitical structures that constitutively frame and condition its exercise, and which SAR helps configure. Thus, the implications of SAR development for healthcare concern the kind of intersubjective relations, power structures and human capabilities that this AI assistive robotic technology contributes to shape and foster. Consequently, the philosophical perspective substantively remaps the current terrain of normative consideration on SAR development with a view to freedom, broadening reflection towards SAR implications for the political-structural dimension of such human capacity. In turn, this brings to the fore of ethical concern the so far overlooked structural dimension of human–robot relations, thus addressing the political dimension of technology.

## Conclusions

In response to the significant shortcomings of the predominant ethical approach to SAR, this paper has advanced the philosophical idea of freedom as a transversal ethical concept for the normative reflection on technology development. That is, it has been developed as a concept that leads to critically addressing SAR development in terms of its implications at the *micro*, *meso* and *macro* levels of human life, i.e., regarding its disruptive potential for the individual’s life, the (health)care practices, and the sociopolitical structure.

Drawing upon relevant philosophical accounts that gather several constitutive dimensions of this human capacity, this paper has disclosed a feature of freedom that is key for an ethical reflection in the light of this notion, namely its political-structural dimension of freedom. That is, the fact that freedom is constitutively linked to the sociopolitical framework within which it is (to be) exercised, and thus, far from being an individual capacity or exercise, it is related to the sociopolitical structuring of human life. Revealing the proactive role of technology in the configuration of the sociopolitical framework in which individuals’ life takes place, this paper has showed the pertinence of taking this political-structural dimension under the scope of ethical attention on SAR. Making use of the normative breadth of the unfolded philosophical notion, the relevant issues for SAR development with a view to freedom have been reexamined. In this way, this paper has substantially enriched the current discussion on SAR implications for human freedom by broadening the scope beyond the sphere of human–robot interaction (HRI) and bringing to the fore the so far overlooked structural dimension of human–robot relations.

This proposed conceptual framework is not only relevant to SARs but also to other smart assistive technologies and other kinds of robots. However, in this paper we have focused on SARs as a particularly striking case where the rhetoric of empowering users-patients contrasts sharply with the threats to freedom and autonomy that we have identified starting from an ethical–political analysis.

## Footnotes

1 In this paper, the term “(sociopolitical) structure” is used to broadly refer to the institutional background of individuals' relational standing and activity in society (i.e., the political, legal, social and economic order of a society). In this sense, it is a term committed to the Rawlsian concept of "basic structure of society", defined as "[society’s] main political, social and economic institutions and the how they fit together into one unified system of social cooperation from one generation to the next" and defended as the first subject of justice [38], 11).

2 As explained in Sec. 4, this dimension refers to the kind of human involvement with technology that is defined by a direct interaction with or use of a technological artifact or system. The adjectivation “interpersonal” thus is not meant to be literally understood as refering to a relation between two persons, but, at most, between two agents—agency being then an unrefined concept dissociated from morality–. That is, if at all, it is to be taken in the minimalist terms of an “interagentive” relation.

3 Under this conception, impediments to freedom are understood as deliberate interferences, rather than mere contingencies beyond (direct) human control. Thus, uncoerced activity refers here to that activity that one can undertake insofar as it is not obstructed by other people, and not by factors such as natural causes or physical or mental limitations.

4 It would be unlikely to talk about an agent being free were not possible for them to act according with their rational deliberation on the grounds for that action. Such conception would entail to reduce freedom to a mere exercise of reason, and to a condition proper of a *noumenical* subject. In turn, this would leave paternalism outside the scope of what counts as an infringement to freedom as autonomy, had the affected individuals exercised an own rational deliberation on the reasons for action—nonetheless disregarded as a valuable basis for a de facto self-government of these agents.

5 Notice that therewith we do not mistakenly mean “freedom” to be a “capability” according to Nussbaum. Indeed, freedom is not included as such in her list of the ten central human capabilities. Rather, we just aim at highlighting that freedom, as a capacity (to govern oneself), is best read from a Nussbaumian understanding of capacity as “capability”.

6 Such an account has also been labeled as dyadic domination [20]. However, we will refer to it as “intersubjective domination”, since it may better account for the republican conception of domination as possibly held not only by personal (individual) but also corporate/collective agents.

7 Whereas “capabilities” refer to the doings and beings that people can achieve, “functionings” refer to the doings and beings that are realisations of that “capabilities”. As exposed later in Sect. 4, such Nussbaumian distinction between capabilities and functionings is crucial for normatively addressing social robotics development for assistive contexts.

8 Although Nussbaum originally names them “internal capabilities”, following [39] we have chosen to use this alternative terminology to overcome the interpretation problems ascribed to Nussbaum’s one.

9 Notice that, by extension, this role has implications for the broader ethics of technology along the same lines: any normative reflection on technology must always include attention to issues arising from the perspective of justice.

10 See footnote 5.

11 Note that, in line with the conceptual nature of our work, the goal of this Section is to map the kind of issues that should be taken into account for SAR development from a philosophical account of freedom, rather than to engage in a case study of how these typologies of issues are instantiated in a specific care context. Precisely because of the poor understanding of freedom that has so far informed the normative-oriented discussion on SAR, together with the fact that such robots’ implementation remains still in pilot stages, there is a lack of empirically elaborated cases on freedom comprehensively understood. Therefore, the examples provided in this Section are meant to have a secondary role in the analysis, aimed at assisting the conceptual work and helping to making sense of the kind of freedom-challenging phenomena that SAR could raise regarding each dimension of such capacity, within a broad care context. This explains why such examples are posed in a hypothetical tone.

12 This is in line with what has already happened, for example, with the infrastructure of functioning co-created by the extended use of mobile phones, outside of which we cannot easily function regarding significant facets of life anymore, such as work or even healthcare services’ access.

13 See footnote 7.
